# Imaging Features of Retinal Vasculitis and/or Retinal Vascular Occlusion after Brolucizumab Treatment in the Postmarketing Setting

**DOI:** 10.1016/j.xops.2023.100361

**Published:** 2023-07-01

**Authors:** Dilraj S. Grewal, Charles C. Wykoff, Divya D’Souza, Valentine Jehl, Iulian Alecu, Glenn J. Jaffe

**Affiliations:** 1 Department of Ophthalmology, Duke University, Durham, North Carolina; 2 Retina Consultants of Texas, Houston, Texas; 3 Novartis Pharmaceuticals Corp., East Hanover, New Jersey; 4 Novartis Pharma AG, Basel, Switzerland

**Keywords:** Brolucizumab, Imaging, Intraocular inflammation, Retinal vasculitis, Retinal vascular occlusion

## Abstract

**Purpose:**

The aim of this analysis was to characterize the spectrum of inflammatory changes arising from brolucizumab use in routine clinical practice.

**Design:**

Retrospective analysis of fluorescein angiography (FA), fundus photography (FP) and OCT images taken at the time of adverse event.

**Subjects:**

Brolucizumab-treated patients with neovascular age-related macular degeneration with retinal vasculitis (RV) and/or retinal vascular occlusion (RO) reported to Novartis Patient Safety between February 2020 and January 2021.

**Methods:**

Ocular images were reviewed by an external reading center using predefined grading lists for FA, FP, and OCT.

**Main Outcome Measures:**

Classification of images, the most common imaging features of RV and/or RO by each imaging modality, and the anatomical location of the adverse event in relation to the macula.

**Results:**

Gradable images (N = 475; 222 eyes; 198 patients) were classified as RV only (n = 72); RO only (n = 9), RV + RO (n = 63); posterior segment intraocular inflammation (n = 31); or none by imaging (n = 47). Of the 144 eyes with RV and/or RO, the most common imaging features were vascular leakage on FA, perivascular sheathing on FP, and hyperreflective dots in the vitreous humor on OCT. Retinal vascular occlusion was mainly branched and arterial, affecting multiple vessels.

**Conclusions:**

Although no distinct inflammatory phenotype pathognomonic to brolucizumab-related inflammation was identified, this study increases our understanding of the spectrum of posterior segment inflammatory changes that may occur in brolucizumab-treated neovascular age-related macular degeneration patients, highlighting the potential value of widefield retinal imaging and angiography to detect these inflammatory adverse events.

**Financial Disclosure(s):**

Proprietary or commercial disclosure may be found after the references.

Events of occlusive vasculitis have been reported with the use of the anti-VEGF single-chain antibody fragment brolucizumab, and a safety signal of retinal vasculitis (RV) and/or retinal vascular occlusion (RO) with or without presence of intraocular inflammation (IOI) that may result in severe vision loss has been confirmed.^1^, ^2^, ^3^, ^4^, ^5^, ^6^, ^7^, ^8^, ^9^ Prior reports of brolucizumab-related adverse events with images have been limited to small sample sizes comprising case series and individual case reports.^3^^,^^4^^,^^6^^,^^7^ There is a lack of data characterizing the spectrum of changes seen on retinal imaging evaluated in a standardized manner in a large data set; these data would help to better determine the frequency of different abnormalities. Novartis developed an enhanced pharmacovigilance program to enable better characterization of these adverse events in the brolucizumab postmarketing setting, including a better understanding of the spectrum of imaging features on the various imaging modalities used to detect and evaluate these events. The enhanced pharmacovigilance program included the collection of ocular images and a review of these images in a standardized manner by an independent reading center. Grading lists were developed for different imaging modalities including fluorescein angiography (FA), fundus photography (FP), indocyanine green angiography, OCT, and OCT angiography. These lists were based on changes reported in the literature after brolucizumab use as well as changes expected to be seen in eyes with IOI.

Here, we provide an overview of the features obtained from images collected at the time of adverse events in patients with neovascular age-related macular degeneration and reported to Novartis Patient Safety. The aim of the study was to describe the spectrum and distribution of imaging findings based on a standardized reading center review of eyes to improve the understanding of the most frequently observed imaging features of RV and/or RO in neovascular age-related macular degeneration patients treated with brolucizumab in routine clinical practice.

## Methods

### Study Design

This was a noninterventional descriptive study aimed at improving the understanding of the most common imaging features obtained from images collected at the time of RV/RO events. Whenever such adverse events pertaining to RV and/or RO were reported to Novartis Patient Safety, the reporter was encouraged to share all available ocular images obtained as part of standard clinical practice. Ocular images shared with Novartis until January 31, 2021, from all countries where brolucizumab is approved and used per routine clinical practice, were considered for this study. Although the emphasis of the ocular image collection was on cases identified as RV and/or RO by the treating physician in the pharmacovigilance system, cases where the treating physician provided images of eyes with IOI were also included in the analysis. This study complied with the tenets of the Declaration of Helsinki and is based on a retrospective analysis of deidentified data obtained as part of routine clinical practice. As the patient data was spontaneously reported to the pharmacovigilance data system, consent was considered implicit and specific institutional review board approval was not required. Duke Reading Centre has institutional review board approval to conduct research on all deidentified ocular images submitted to the reading center (Duke IRB Pro00046442). Additional consent was obtained from the reporting physicians for the utilization of images for publication purposes. Only those images for which both the patients and reporting physicians granted consent have been included in this publication.

The submitted images were reviewed by an expert reader who was an experienced vitreoretinal and uveitis specialist (D.G.) at an external reading center (Duke Reading Center, Durham, NC [referred to as the “reading center” hereafter]). Predefined grading variable lists for evaluating inflammatory and ischemic signs were developed for each of the imaging modalities: FA, FP, indocyanine green angiography, OCT, and OCT angiography (Table 1). Criteria used to evaluate the presence, extent, and location of findings are listed in Table S2 (available at www.ophthalmologyscience.org). No restriction was made on the type of images collected or the method of capture (i.e., any image type acquired on any platform could be shared by the treating physician).

**Table 1:** Assessed Imaging Variables by Imaging Modality

FP	FA	OCT	OCTA	ICGA
Perivascular sheathing	Retinal arterial occlusion	Vitreous/preretinal hyperreflective dots	Foveal avascular zone perimeter irregularity	Early choroidal vessel hyperfluorescence
Retinal vessel box-carring	Retinal vein occlusion	Inner retinal layer hyperreflectivity	Superficial capillary plexus ischemia	Choroidal hypofluorescent areas
Retinal whitening	Vascular leakage	Paracentral acute middle maculopathy	Deep capillary plexus ischemia	Optic nerve head hyperfluorescence
Cotton wool spots	Retinal ischemia	Cystoid macular edema	Choriocapillaris ischemia	
Kyrieleis plaques	Optic nerve head hyperfluorescence	Retinal nerve fiber layer edema around optic nerve		
Retinal hemorrhage	Retinal vessel box-carring	Inner retinal thinning		
Optic nerve swelling	Retinal neovascularization			
Media opacities	Choroidal hypoperfusion (early phase)			
Vitreous hemorrhage				

FA = fluorescein angiography; FP = fundus photography; ICGA = indocyanine green angiography; OCTA = OCT angiography.

For each reviewed case, the reading center classified the eye based on all provided imaging data acquired at the time of event into 1 of 6 categories: “RV only,” “RO only,” “RV + RO only,” “posterior segment IOI only,” “none by imaging,” or “not assessable.” This categorization was based on the evaluation of posterior segment inflammation on the available images only, including for IOI. Classification criteria for the different imaging modalities are presented in Table S3 (available at www.ophthalmologyscience.org)**.** Anatomical location in relation to the macula (i.e., macula [the region of the retina within the major temporal vascular arcades] vs. midperiphery [the region of the retina extending from the vascular arcades to the posterior edge of the vortex vein ampulla] vs. periphery [the region of the retina anterior to the vortex vein ampulla]) and location details were summarized by eye case classification based on imaging data.^10^ For each submitted case, time point, and imaging modality, the full assessment included a notation as to whether the images for each modality were of adequate quality to be gradable, and the pathological features identified on each image. Only images from the treated and affected eye, taken at the time of event, were included in the analyses.

Given the incompleteness of information submitted through postmarketing reporting, best-corrected visual acuity (BCVA) data could only be analyzed from a subgroup of patients in Japan. The pre-event BCVA was defined as the BCVA documented closest in time before the event (documentation of inflammation by the treating physician) and the postevent BCVA was defined as the latest BCVA documented after the event.

### Statistical Analysis

All analyses were descriptive in nature. Anatomical location, sublocation, and type of occlusion in the eye were summarized based on imaging data by imaging modality. The extent of involvement was also determined. Each patient’s eyes were assessed independently.

## Results

### Image Classification by the Reading Center

Overall, a total of 484 image sets (a set included all the images from a particular imaging modality, FP, FA, and OCT) from the time of a reported adverse event for 231 brolucizumab-treated and affected eyes were reviewed by the reading center. Image sets of 9 eyes were not gradable because of poor image quality. Accordingly, there were a total of 475 gradable image sets of 222 eyes from 198 patients that were included in the study cohort.

Demographic information is summarized in Table 4. Patients were mostly ≥ 70 years of age, and the majority were female (103/198, 52%), compared with males (90/198, 45%); gender was missing for 3% of the patients (5/198). Most of the eyes with RV and/or RO were reported from Japan (50%) and the United States (41%). The most common gradable images provided were FP (n = 172), OCT (n = 166) and FA (n = 105) (Table 5). Eighteen of the 105 fluorescein angiograms, 27 of the 172 fundus photographs, and 2 of the 20 indocyanine green angiograms reviewed were widefield.

**Table 4:** Patient Characteristics in Case Cohort with Gradable Images

	IOI n = 31	RV only n = 65	RO only n = 8	RV+RO only n = 60	RV and/or RO n = 133	None n = 34
Age (yrs), n (%)						
50-< 60	1 (3)	1 (2)	0 (0)	2 (3)	3 (2)	0 (0)
60-< 70	3 (10)	14 (22)	0 (0)	2 (3)	16 (12)	7 (21)
70-< 80	15 (48)	23 (35)	5 (63)	25 (42)	53 (40)	11 (32)
≥ 80	9 (29)	19 (29)	3 (38)	27 (45)	49 (37)	8 (24)
Age unknown	2 (6)	4 (6)	0 (0)	1 (2)	5 (4)	5 (15)
Missing	1 (3)	4 (6)	0 (0)	3 (5)	7 (5)	3 (9)
**Gender, n (%)**						
Female	17 (55)	27 (42)	5 (63)	42 (70)	74 (56)	12 (35)
Male	14 (45)	37 (57)	3 (38)	17 (28)	57 (43)	19 (56)
Missing	0 (0)	1 (2)	0 (0)	1 (2)	2 (2)	3 (9)
**Country, n (%)**						
Australia	2 (6)	1 (2)	0 (0)	0 (0)	1 (1)	1 (3)
Canada	1 (3)	0 (0)	0 (0)	1 (2)	1 (1)	0 (0)
Germany	1 (3)	2 (3)	1 (13)	2 (3)	5 (4)	5 (15)
Japan	23 (74)	46 (71)	2 (25)	18 (30)	66 (50)	14 (41)
Malaysia	0 (0)	1 (2)	0 (0)	0 (0)	1 (1)	0 (0)
Portugal	1 (3)	0 (0)	0 (0)	2 (3)	2 (2)	0 (0)
Switzerland	1 (3)	1 (2)	0 (0)	0 (0)	1 (1)	1 (3)
United Arab Emirates	0 (0)	1 (2)	0 (0)	0 (0)	1 (1)	0 (0)
United States	2 (6)	13 (20)	5 (63)	37 (62)	55 (41)	13 (38)

Number of patients = 198. Patients with bilateral events are counted under the worse-eye classification considering the following hierarchy: None < IOI < RV only < RO only < RV+RO only.

IOI = intraocular inflammation; RO = retinal vascular occlusion; RV = retinal vasculitis.

**Table 5:** Distribution of 475 Gradable Images Across all Available Imaging

Imaging Modality	Total Gradable (Number of Images)	Widefield, Number (%)
Any modality (number of images)	475	
FA	105	18 (17)
FP	172	27 (16)
OCT	166	–
OCTA	12	–
ICGA	20	2 (10)

FA = fluorescein angiography; FP = fundus photography; ICGA = indocyanine green angiography; OCTA = OCT angiography.

**Figure 1:**
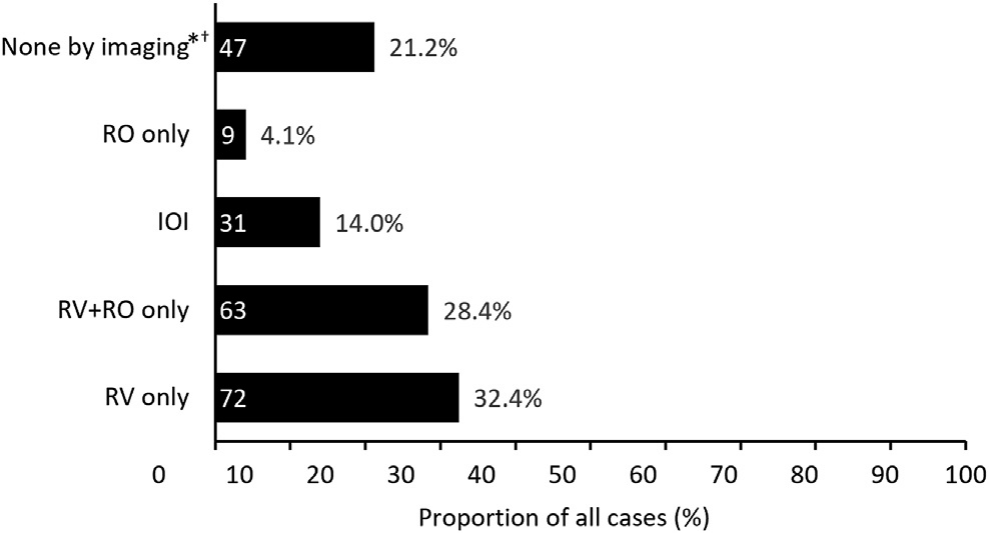
Number of eyes inside bar base; proportion (%) of all 222 eyes at bar tip. ∗Only signs of posterior segment inflammation were investigated. †A total of 33/47 eyes (70.2%) classified as “none by imaging” by the reading center were reported as anterior segment intraocular inflammation (IOI) by the initial reporter in the pharmacovigilance system. RO = retinal vascular occlusion; RV = retinal vasculitis.

Based on gradable images from the 222 collected eyes, the reading center classified 32.4% (72/222) as RV only; 28.4% (63/222) as RV + RO only; 14.0% (31/222) as posterior segment IOI; 4.1% (9/222) as RO only; and 21.2% (47/222) as "none by imaging" (Fig 1).

### Most Common Imaging Features and Features of Inflammation Seen on FA, FP, and OCT

Among the 144 eyes with RV and/or RO, abnormalities were seen on FA in 79 eyes, on FP in 126 eyes, and on OCT in 52 eyes. The most common abnormal FA features were vascular leakage (71/79 eyes [89.9%]), retinal arterial occlusion (53/79 eyes [67.1%]), retinal ischemia (46/79 eyes [58.2%]) and optic nerve head hyperfluorescence (43/79 eyes [54.4%]); the most common abnormal FP features were perivascular sheathing (105/126 eyes [83.3%]), media opacities (58/126 eyes [46.0%]), and retinal hemorrhages (36/126 eyes [28.6%]); and the most common abnormal OCT features were hyperreflective dots in vitreous (33/52 eyes [63.5%]), inner retinal layer hyperreflectivity (19/52 eyes [36.5%]), and paracentral acute middle maculopathy (12/52 eyes [23.1%]; Table 6). Examples of RV and RO imaging features are shown in Figures S2–S4 (available at www.ophthalmologyscience.org).

**Table 6:** Abnormal Imaging Features in Eyes with RV and/or RO Observed on Abnormal FA, FP, and OCT

Modality / Grading variables	RV and/or RO n (%)
**FA**	**n = 79**
Vascular leakage	71 (89.9)
Retinal arterial occlusion	53 (67.1)
Retinal ischemia	46 (58.2)
Optic nerve head hyperfluorescence	43 (54.4)
Retinal vessel box-carring	9 (11.4)
Retinal vein occlusion	5 (6.3)
Choroidal hypoperfusion (early phase)	3 (3.8)
**FP**	**n = 126**
Perivascular sheathing	105 (83.3)
Media opacities	58 (46.0)
Retinal hemorrhages	36 (28.6)
Retinal whitening	29 (23.0)
Cotton wool spots	27 (21.4)
Kyrieleis plaques	19 (15.1)
Retinal vessel box-carring	3 (2.4)
Optic nerve swelling	2 (1.6)
Vitreous hemorrhage	1 (0.8)
**OCT**	**n = 52**
Hyperreflective dots in vitreous	33 (63.5)
Inner retinal layer hyperreflectivity	19 (36.5)
Paracentral acute middle maculopathy	12 (23.1)
Retinal nerve fiber layer edema around optic nerve	8 (15.4)
Inner retinal thinning	2 (3.8)

FA = fluorescein angiography; FP = fundus photography ; RO = retinal vascular occlusion; RV = retinal vasculitis.

% for “Grading variable” = number of images with grading variable/total number of images with this imaging modality abnormality per independent classification.

### Frequency and Location of Imaging Abnormalities in Relation to the Macula and Effects on Visual Acuity

Most eyes with RV and/or RO had macular involvement, but in 14% of eyes (20/144) the midperiphery and/or periphery was affected **(**Table 7**).** For RV + RO-only cases, the retinal occlusion was mainly branch rather than central; peripheral vessels were involved in about a quarter of cases and multiple vessels were more likely to be involved than single vessels. Occlusions were mainly arterial (53 cases); however, there were 5 cases that also had venous occlusion **(**Table 8**)**.

**Table 7:** Frequency of Anatomical Location in Relation to the Macula (n = 144)

Anatomical Location in Relation to Macula	RV and/or RO n (%) eyes
Macula only	41 (28.5)
Macula + midperiphery + periphery	44 (30.6)
Macula + midperiphery	37 (25.7)
Midperiphery + periphery	7 (4.9)
Midperiphery only	12 (8.3)
Periphery only	1 (0.7)

RO = retinal vascular occlusion; RV = retinal vasculitis.

**Table 8:** Extent and Location of Retinal Arterial and Vein Occlusions in Eyes with Arterial and Vein Involvement

	RV only	RO only	RV + RO only	RV and/or RO
Arterial	n = 57	n = 3	n = 60	n = 120
Retinal arterial occlusion, n (%)	0	3 (100)	50 (83)	53 (44)
Type				
Central	0	1	9	10
Branch	0	2	27	29
Peripheral	0	0	13	13
Number				
Single	0	3	11	14
Multiple	0	0	37	37
Vein	n = 5	n = 0	n = 8	n = 13
Retinal vein occlusion, n (%)	0	0	5 (63)	5 (38)
Type				
Branch	0	0	3	3
Peripheral	0	0	2	2
Number				
Single	0	0	2	2
Multiple	0	0	3	3

Data from some eyes are missing as not all characteristics were gradable or available.

RO = retinal vascular occlusion; RV = retinal vasculitis.

In patients with paired BCVA event data (i.e., BCVA before the event vs. last available BCVA after the event; n = 50), the median Snellen BCVA before the event was 20/80 (range, 20/21–20/632; 0.60 logarithm of the minimum angle of resolution [range, 0.02–1.5]), and the latest Snellen BCVA after the event was 20/63 (range, 20/21–20/632; 0.50 logarithm of the minimum angle of resolution [range, 0.03–1.5]). The mean BCVA change after versus before the event was 0.07 logarithm of the minimum angle of resolution (*P* = 0.052).

## Discussion

The aim of this study was to gain a better understanding of the most observed imaging features obtained from images collected at the time of RV/RO events and reported to Novartis Patient Safety. Although this is the largest descriptive analysis to date of imaging features associated with brolucizumab-related IOI, taken at the time of event occurrence, it is important to recognize that this is not an incidence study. Most eyes were classified as RV only, followed by RV + RO only, and then posterior segment IOI. This outcome is expected as the focus and main efforts of the data collection was on adverse events of RV and/or RO. For events reported as IOI by the reporting physician, the images were not actively requested in the case documentation process; however, in some cases these were spontaneously submitted by the reporter.

In eyes with RV and RV + RO, the most common imaging abnormalities were vascular leakage on FA, perivascular sheathing on FP, and hyperreflective dots in the vitreous on OCT. Retinal vascular occlusion was mainly arterial; more often branch than central; peripheral vessels were involved in about one quarter of eyes; and multiple rather than single vessels were more likely to be involved. Although widefield imaging was not obtained across all eyes, in 14% of eyes only the midperiphery and/or periphery was affected, and the macula was spared. This suggests a potential value of widefield retinal imaging, particularly widefield FA, for the detection of changes in the periphery. In a small subset of eyes with available visual acuity measurements (n = 50), we did not identify an overall mean decrease in visual acuity compared with visual acuity before the events.

Key strengths of this study are the standardized interpretation of the collected images leading to a standardized imaging-based classification of anatomical features of the retina. Key limitations of this study include factors inherent to postmarketing reporting systems such as underreporting, incomplete documentation of adverse event cases, incomplete clinical and demographic information, lack of standardization of imaging, and eyes without adequate imaging information. Furthermore, as postmarketing adverse event reporting is voluntary, selective reporting cannot be excluded, and results may not be representative of all treated eyes. In particular, IOI cases are underrepresented because ocular images were not specifically sought for cases reported as IOI in the pharmacovigilance system. Considering the context of this study, this data set may also be subject to ascertainment bias typical of postmarketing voluntary reporting, with events reported being more severe in nature. We also acknowledge that the population of patients after a drug is launched may be very different from the population studied in the premarketing phase of a drug's approval.

Reporting practices vary by country, physician, the type of event, and the visual outcomes, and ocular images were not always captured at the time of event. Moreover, due to the nature of the study, all images provided by the treating physician were accepted and there was no standardized image acquisition protocol; accordingly, midperipheral and peripheral images and multi-imaging modalities were not available for all eyes. Also, because the vast majority of images provided were those taken at the time of event, it was not possible to determine features predictive of adverse events.

The objective of this paper is not to define reading center-based image grading techniques or to provide consensus definitions for various retinal vascular events seen after intravitreal brolucizumab but rather to share the observations made for the events for which images were available to illustrate the possible characteristics of the event. We acknowledge that the features described in this paper may not represent the full spectrum of possible adverse events seen after intravitreal brolucizumab and this data set only represents the cases that were captured using a voluntary postmarketing pharmacovigilance reporting system.

In summary, we identified a diverse range of location and severity of inflammatory involvement in this data set, and it is important for physicians to recognize this heterogeneous spectrum of involvement. The data also suggest that there may not be a distinct inflammatory phenotype that is pathognomonic to brolucizumab-related inflammation. These findings increase our understanding of the spectrum of posterior segment inflammatory changes occurring in eyes treated with brolucizumab and may further guide clinical practice by informing physicians on imaging features indicative of an inflammatory event. Additionally, this study also highlights the potential value of widefield retinal imaging and angiography to detect these adverse events.
