# Supplementary material for: Imaging Features of Retinal Vasculitis and/or Retinal Vascular Occlusion after Brolucizumab Treatment in the Postmarketing Setting
Source: Ophthalmol Sci. 2023 Jul 1;4(1):100361. doi: 10.1016/j.xops.2023.100361 (PMC10587630; doi:10.1016/j.xops.2023.100361)

Supplemental Figure S1. Examples of imaging features in retinal vasculitis.
A) Cotton wool spot (white arrow) in the posterior pole and a kyrieleis plaque (red arrow) in the posterior pole along the inferior arteriole (blue arrow). Image courtesy of Dr. Alex Casanova. **B)** Perivascular leakage in the posterior pole along the inferior arteriole. Image courtesy of Dr. Alex Casanova. **C)** Branch retinal artery occlusion with retinal ischemia in the macula (orange arrow). Image courtesy of Dr. Vishak John.

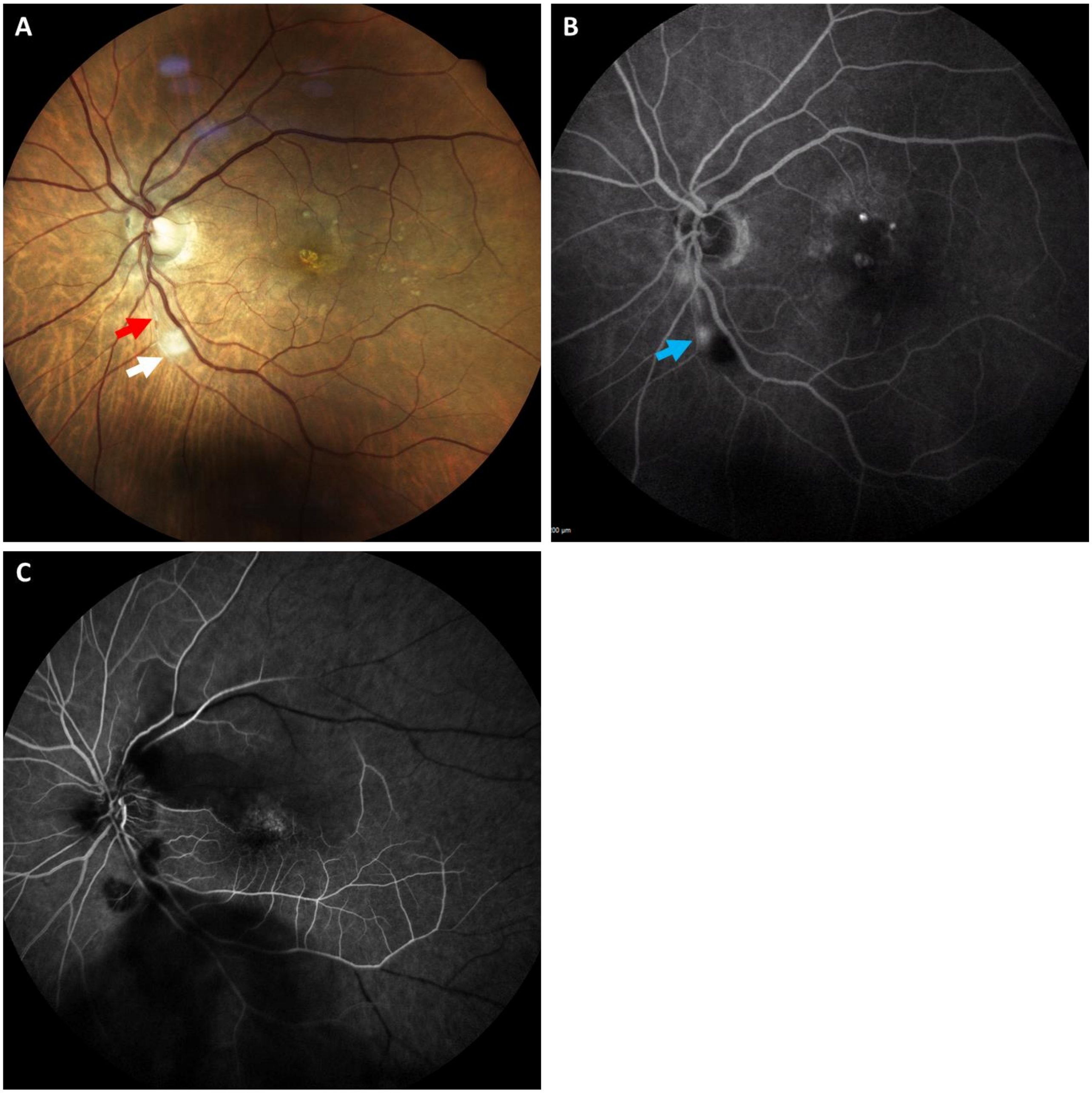

Supplement: Figure S2 [file mmc1.pdf]
