# Supplementary material for: Imaging Features of Retinal Vasculitis and/or Retinal Vascular Occlusion after Brolucizumab Treatment in the Postmarketing Setting
Source: Ophthalmol Sci. 2023 Jul 1;4(1):100361. doi: 10.1016/j.xops.2023.100361 (PMC10587630; doi:10.1016/j.xops.2023.100361)

Supplemental Figure S2. A case of retinal vasculitis.
A) Arterial perivascular sheathing (green arrow), retinal whitening in macula (blue arrow) and kyrieleis plaques (red arrows). **B)** Fluorescein angiogram showing vascular leakage in macula (white arrow). **C)** OCT showing vitreous hyper-reflective dots (yellow arrow). Images courtesy of Dr. Sumiyo Noge.

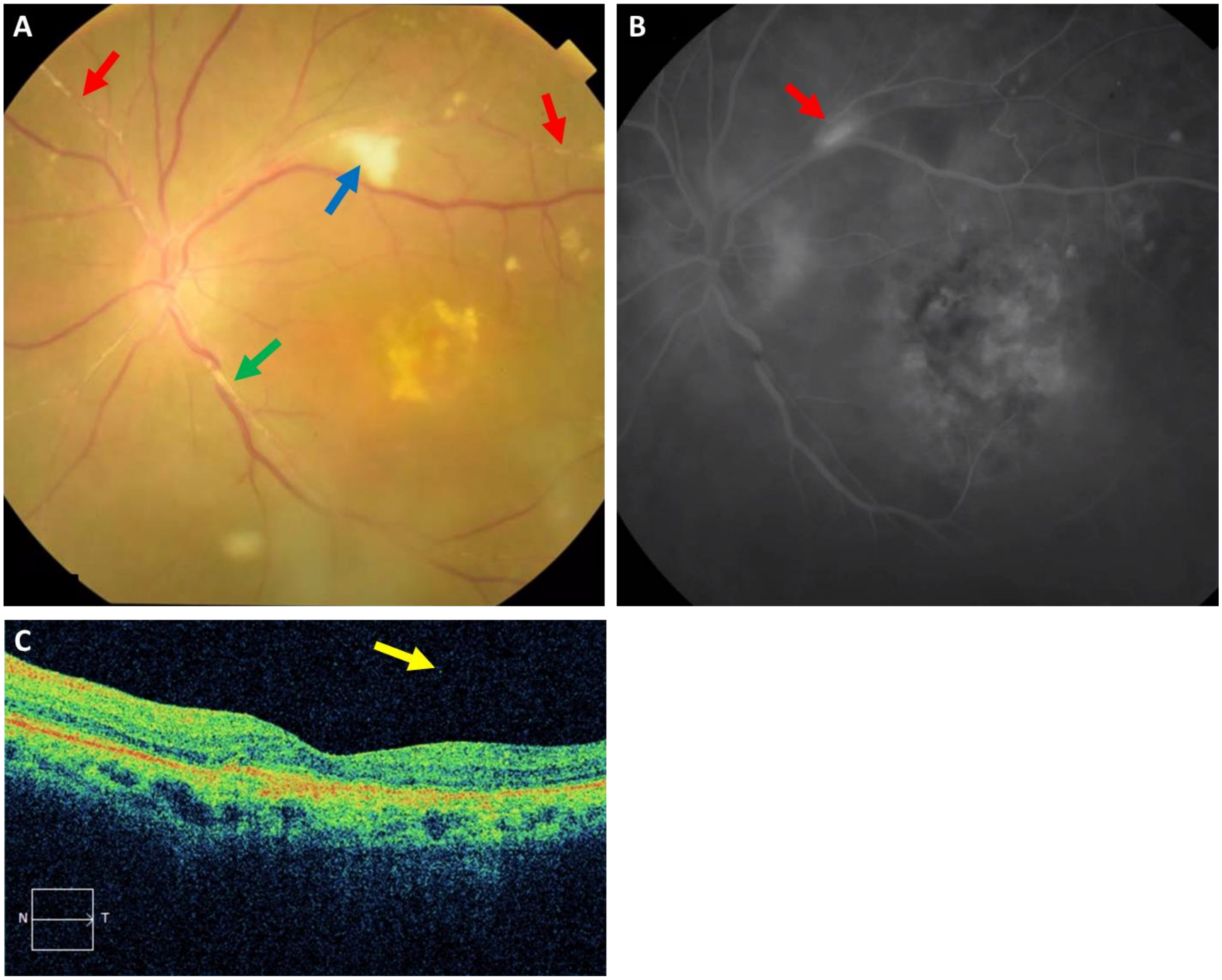

Supplement: Figure S3 [file mmc2.pdf]
