# Supplementary material for: Imaging Features of Retinal Vasculitis and/or Retinal Vascular Occlusion after Brolucizumab Treatment in the Postmarketing Setting
Source: Ophthalmol Sci. 2023 Jul 1;4(1):100361. doi: 10.1016/j.xops.2023.100361 (PMC10587630; doi:10.1016/j.xops.2023.100361)

Supplemental Figure S3. A case of retinal vasculitis.

A) Retinal whitening (blue arrows) kyrieleis plaque (green arrows) and vitreous opacities (white arrows). **B)** OCT showing vitreous hyper-reflective dots (yellow arrow). **C)** On follow-up, there is resolution of retinal whitening and reduction in kyrieleis plaques. D) OCT shows resolution of vitreous hyper-reflective dots. Images courtesy of Dr. Rumiko Hara.

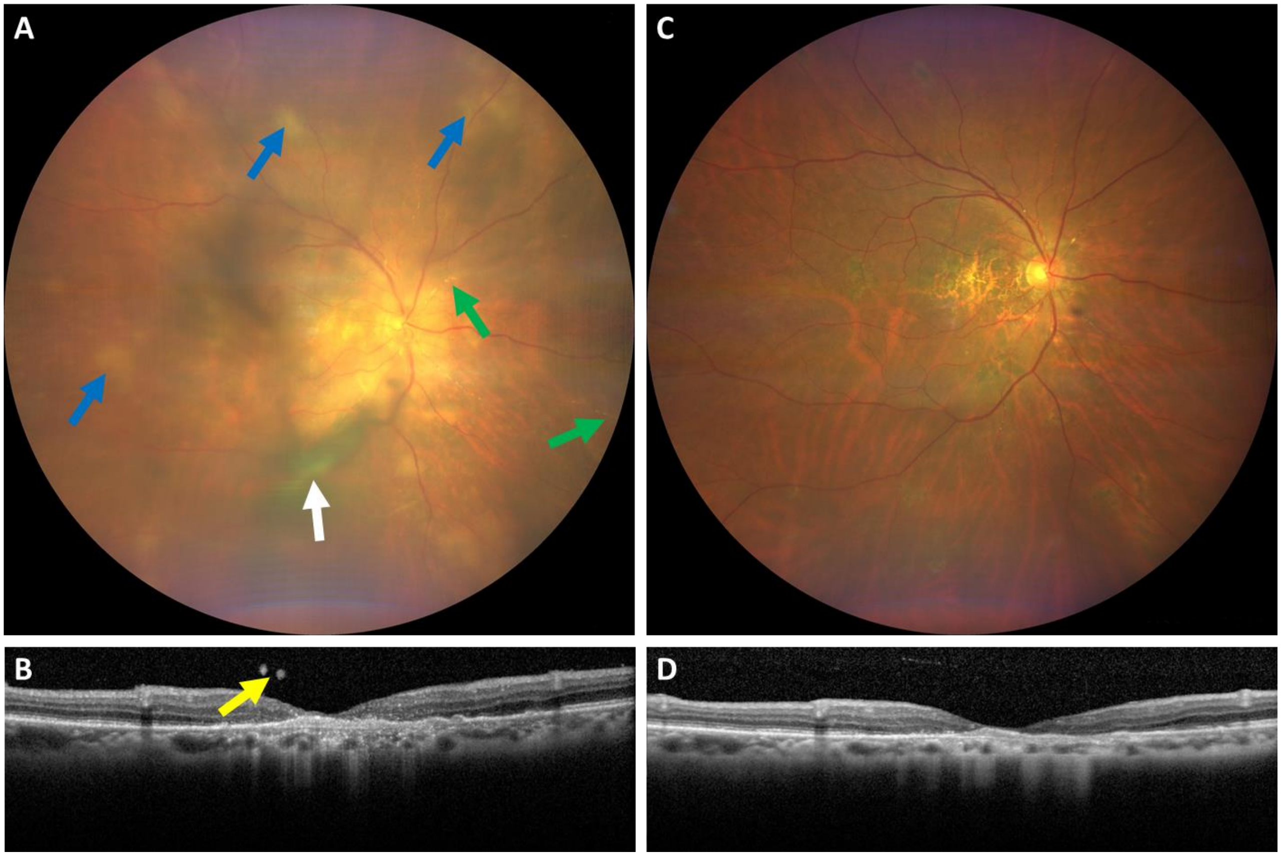

Supplement: Figure S4 [file mmc3.pdf]
