# Supplementary material for: Imaging Features of Retinal Vasculitis and/or Retinal Vascular Occlusion after Brolucizumab Treatment in the Postmarketing Setting
Source: Ophthalmol Sci. 2023 Jul 1;4(1):100361. doi: 10.1016/j.xops.2023.100361 (PMC10587630; doi:10.1016/j.xops.2023.100361)
Supplement: Table S1 [file mmc4.pdf]

Supplemental Table S1. Determination of anatomical location in relation to the macula by image modality

Anatomical location in relation to the macula	Image modality	Location detail - Definition/ item on check list
**Macula**	FA or FP	Retinal Vessel Box-carring
	FA	Vascular Leakage
	FA	Retinal Ischemia
	FA	Retinal Neovascularization
	FP	Perivascular Sheathing
	FP	Retinal Whitening
	FP	Cotton-wool Spots
	FP	Kyrieleis Plaques
	FP	Retinal Hemorrhages
	ICGA	Choroidal Hypocyanescent Areas
	OCT or OCT-A	Include all features
**Mid-periphery**	FA or FP	Retinal Vessel Box-carring
	FA	Vascular Leakage
	FA	Retinal Ischemia
	FA	Retinal Neovascularization
	FP	Perivascular Sheathing
	FP	Retinal Whitening
	FP	Cotton-wool Spots
	FP	Kyrieleis Plaques
	FP	Retinal Hemorrhages
	ICGA	Choroidal Hypocyanescent Areas
**Periphery**	FA	Retinal Arterial Occlusion
	FA	Retinal Vein Occlusion
	FA or FP	Retinal Vessel Box-carring
	FA	Vascular Leakage
	FA	Retinal Ischemia
	FA	Retinal Neovascularization
	FP	Perivascular Sheathing
	FP	Retinal Whitening
	FP	Cotton-wool Spots
	FP	Kyrieleis Plaques
	FP	Retinal Hemorrhages
	ICGA	Choroidal Hypocyanescent Areas

FA, fluorescein angiography; FP, fundus photography; ICGA, indocyanine green angiography; OCT, optical coherence tomography; OCT-A, optical coherence tomography angiography.
