# Supplementary material for: Imaging Features of Retinal Vasculitis and/or Retinal Vascular Occlusion after Brolucizumab Treatment in the Postmarketing Setting
Source: Ophthalmol Sci. 2023 Jul 1;4(1):100361. doi: 10.1016/j.xops.2023.100361 (PMC10587630; doi:10.1016/j.xops.2023.100361)
Supplement: Table S2 [file mmc5.pdf]

Supplemental Table S2. Imaging features required for IOI, RO, RV classified by image modality

Pathology	Imaging modality	Imaging feature
RV	FP:	Perivascular sheathing, Kyrieleis plaque
	FA:	Vascular leakage
	ICG:	Early choroidal vessel hypercyanescence
RO	FP:	Retinal vessel box-carring
	FA:	Retinal arterial occlusion, retinal vein occlusion, retinal vessel box-carring, retinal ischemia, retinal neovascularization
	ICG:	Choroidal hypocyanescent areas
	OCT:	Inner retinal layer hyperreflectivity, paracentral acute middle maculopathy
	OCT-A:	Superficial or deep capillary plexus ischemia
IOI*	FP:	Media opacities
	OCT:	Vitreous hyper-reflective dots
None by imaging	All	No imaging features of IOI, RV, or RO
Not assessable	All	Poor image quality prevents grading

*Case classification based on absence of imaging evidence of RO or RV.

FA, fluorescein angiography; FP, fundus photography; ICGA, indocyanine green angiography; IOI, intraocular inflammation; OCT, optical coherence tomography; OCT-A, optical coherence tomography angiography; RO, retinal vascular occlusion; RV, retinal vasculitis.
